# Characterizing asparagine synthetase deficiency variants in lymphoblastoid cell lines

**DOI:** 10.1002/jmd2.12356

**Published:** 2023-01-05

**Authors:** Stephen J. Staklinski, Mario C. Chang, Rebecca C. Ahrens‐Nicklas, Shagun Kaur, Arianna K. Stefanatos, Elizabeth E. Dudenhausen, Matthew E. Merritt, Michael S. Kilberg

**Affiliations:** ^1^ Department of Biochemistry and Molecular Biology University of Florida College of Medicine Gainesville Florida USA; ^2^ School of Biological Sciences Cold Spring Harbor Laboratory, Cold Spring Harbor New York New York USA; ^3^ Division of Human Genetics, Department of Pediatrics Children's Hospital of Philadelphia Philadelphia Pennsylvania USA; ^4^ Department of Child and Adolescent Psychiatry and Behavioral Sciences Children's Hospital of Philadelphia Philadelphia Pennsylvania USA

**Keywords:** amino acids, epilepsy, inborn errors, metabolism, microcephaly

## Abstract

Asparagine synthetase (ASNS) catalyzes the synthesis of asparagine (Asn) from aspartate and glutamine. Biallelic mutations in the *ASNS* gene result in ASNS Deficiency (ASNSD). Children with ASNSD exhibit congenital microcephaly, epileptic‐like seizures, and continued brain atrophy, often leading to premature mortality. This report describes a 4‐year‐old male with global developmental delay and seizures with two novel mutations in the *ASNS* gene, c.614A > C (maternal) and c.1192dupT (paternal) encoding p.H205P and p.Y398Lfs*4 variants, respectively. We employed the novel use of immortalized lymphoblastoid cell lines (LCL) to show that the proliferation of the heterozygotic parental LCL was not severely affected by culture in Asn‐free medium, but growth of the child's cells was suppressed by about 50%. Asn production by the LCL from both the father and the child was significantly decreased relative to the mother's cells. mRNA and protein analysis of the paternal LCL cells for the Y398Lfs*4 variant revealed reductions in both. Attempts to ectopically express the truncated Y398Lfs*4 variant in either HEK293T or ASNS‐null cells resulted in little or no detectable protein. Expression and purification of the H205P variant from HEK293T cells revealed enzymatic activity similar to wild‐type ASNS. Stable expression of WT ASNS rescued the growth of ASNS‐null JRS cells in Asn‐free medium and the H205P variant was only slightly less effective. However, the Y398Lfs*4 variant appeared to be unstable in JRS cells. These results indicate that co‐expression of the H205P and Y398Lfs*4 variants leads to a significant reduction in Asn synthesis and cellular growth.

**SYNOPSIS**

A child with asparagine synthetase deficiency has biallelic mutations encoding H205P and Y398Lfs*4 variants, which lead to a significant reduction in Asn synthesis and cellular growth.

## 1 INTRODUCTION

In humans, asparagine synthetase (ASNS) is the only known mammalian enzyme that catalyzes de novo synthesis of asparagine (Asn), using glutamine as the ammonia donor in an amidotransferase reaction that converts aspartate to Asn.^1^, ^2^ The human *ASNS* gene resides within 35 kb at chromosome 7q21.3 and contains 13 exons that are translated into 561 amino acids to form the 64 kDa functional enzyme.^3^, ^4^, ^5^, ^6^ Human ASNS and *Escherichia coli* AS‐B proteins share 40% sequence identity and the crystal structure of human ASNS^6^ revealed a great deal of similarity when compared with that of AS‐B.^1^, ^2^, ^7^ The ASNS structure consists of distinct N‐terminal (residues 1–208) and C‐terminal (residues 209–561) domains. It is hypothesized that binding of glutamine in the N‐terminus is oriented so that glutaminase activity generates a free ammonia that diffuses through an intramolecular tunnel to the C‐terminus domain, in which the binding of ATP and aspartate leads to an enzyme‐bound β‐aspartyl‐AMP intermediate and pyrophosphate.^2^, ^6^, ^7^, ^8^ Attack of the ammonia on the β‐aspartyl‐AMP intermediate completes the synthesis of Asn and glutamate.

The transcriptional regulation of the *ASNS* gene has been investigated as a component of the cellular response to metabolic stresses including amino acid deprivation and endoplasmic reticulum (ER) stress.^9^, ^10^ In addition, it has been implicated as a promoting factor in a wide variety of cancers and cancer metastasis.^11^ Since 2013, an additional focus of ASNS‐associated research has centered on the inborn error of metabolism resulting from mutations in the *ASNS* gene. The first report of Asn synthetase deficiency (ASNSD) described four families with mutations in the *ASNS* gene.^12^ To date, about 75 ASNSD patients in 55 distinct families have been described.^10^, ^12^, ^13^, ^14^, ^15^, ^16^, ^17^, ^18^, ^19^, ^20^, ^21^ Case reports have commonly described newborns with congenital microcephaly, simplified gyral pattern, axial hypotonia, appendicular spasticity, early onset seizures, and progressive brain atrophy, often leading to premature mortality. Suspicion of ASNSD is typically prompted by recognition of the phenotype in newborns and subsequently confirmed by whole‐exome DNA sequencing that identified mutations in the *ASNS* gene.

The current research arose from identification of a compound heterozygous child with inherited mutations c.614A > C (maternal) and c.1192dupT (paternal) in the *ASNS* gene leading to p.H205P and p.Y398Lfs*4 variant expression. These studies document the biochemical and cellular consequences using protein modeling, purification of variant proteins, and growth analysis using immortalized, cultured cells from the child and parents. Although our previous ASNSD reports involved patient primary fibroblasts in culture,^22^, ^23^, ^24^ the present investigation utilizes the novel approach of immortalized B‐lymphocytes to provide ASNSD lymphoblastoid cell lines (LCL) as a cell culture model. To establish the physiological consequences of each variant in a genetically uniform background and independent of expression of wild‐type (WT) protein or another variant, each variant protein was also ectopically expressed and characterized in an ASNS‐null cell line.

## 2 MATERIALS AND METHODS

### 2.1 Genetic analysis

Buccal swab and/or peripheral blood samples were collected from the patient and sent to a commercial laboratory (GeneDx, Gaithersburg, Maryland) for multigene panel testing. Next‐generation sequencing (NGS) was performed on an Illumina platform and the reads were aligned against the National Center for Biotechnology Information (NCBI) hg19 reference human genome. Variants from the NGS panel were filtered and analyzed using the XomeAnalyzer tool as previously published^25^ and classified according to the American College of Medical Genetics and Genomics/Association for Molecular Pathology (ACMG/AMP) guidelines. Using NGS, 100% of the coding region of the *ASNS* gene was covered at a read depth of at least 10×. Written consent from the parents was obtained prior to collecting the samples, case material, analyzing data, and writing of the final article.

### 2.2 Protein modeling

The human ASNS crystal structure (Uniprot ID: P08243) (PDB: 6GQ3)^6^ was used as a template in UCSF Chimera software (version 1.15) and overlayed with the *Escherichia coli* AS‐B structure (PDB: 1CT9)^7^ to allow modeling based on the previously identified location of bound AMP in the *E. coli* AS‐B crystal.^7^ The H205P variant was modeled with a rotamers tool based on the highest probability backbone‐dependent rotamer library predictions.^26^ Predictions of hydrogen bonding were performed with the FindHBond tool set to default H bond constraints and an evaluation of steric interference was obtained through the Find Clashes/Contacts tool set to parameters for default clash criteria in the Chimera software. Automatic predictions in Chimera were confirmed by manual structure manipulation and the measurement of atom distances. The Y398Lfs*4 variant was modeled similarly but there is a significant amount of ASNS protein structure lost because of the early stop codon introduced by the frameshift mutation.

### 2.3 Cell culture and media conditions

B‐lymphocytes were isolated from blood samples of the affected compound heterozygotic child (p.H205P and p.Y398Lfs*4 ASNS variants) as well as from the mother (p.H205P) and father (p.Y398Lfs*4) by ficol‐gradient centrifugation. Primary patient cell lines were then immortalized with Epstein Bar Virus (EBV) at the Children's Hospital of Philadelphia using standard, previously published methods.^27^, ^28^ An EBV‐immortalized human B‐lymphoblast cell line was obtained from Cellero (no. 1038‐2750JA15) and serves as an unrelated, WT control. These LCL were cultured in RPMI 1640 with L‐glutamine (Corning 10‐040‐CV) supplemented with 15% fetal bovine serum (FBS, Bio‐Techne no. S11550), 1× Penicillin/Streptomycin (Corning no. 30‐002‐CI), and 2 mM additional L‐glutamine. For Asn deprivation experiments, a custom batch of RPMI 1640 lacking L‐Asn was purchased from Corning. HEK293T cells (no. RL‐3216) and ASNS‐null Jensen Rat Sarcoma (JRS) cells (no. CCL‐45) were obtained from ATCC and cultured in high glucose Dulbecco's Modified Eagle's Medium (DMEM; Corning no. 10‐013‐CV) supplemented with 10% FBS, ABAM (streptomycin, penicillin G, and amphotericin B), 1× nonessential amino acids (Corning, no. 25‐025‐CL), and 2 mM glutamine. The DMEM and the RPMI media lacking Asn was prepared by supplementing with 10% dialyzed FBS (Bio‐Techne no. S12850), ABAM. For the DMEM–Asn, the 1× nonessential amino acid mixture was replaced with a freshly prepared 100× stock solution of 10 mM glycine, L‐alanine, L‐aspartate, L‐glutamate, L‐proline, and L‐serine. Prior to initiating the experimental protocols, the cell's nutritional basal state was obtained by providing fresh DMEM medium 12–18 h before all experiments. For all time course cell treatments, the appropriate media were changed every 24 h, a complete DMEM replacement for adherent cells (HEK293T and JRS), and a 50% RPMI replacement for suspension LCL cells to avoid repeated pelleting of the cells.

### 2.4 RNA isolation and quantitative real‐time PCR

Total cellular RNA was isolated and analyzed for specific mRNA species by quantitative real‐time PCR (qRT‐PCR) as described.^24^ Primers used for glyceraldehyde‐3‐phosphate dehydrogenase (GAPDH) mRNA were: forward 5′‐TTGGTATCGTGGAAGGACTC‐3′ and reverse 5′‐ACAGTCTTCTGGGTGGCAGT‐3′. Primers used for ASNS mRNA were: forward 5′‐GCAGCTGAAAGAAGCCCAAGT‐3′ and reverse 5′‐TGTCTTCCATGCCAATTGCA‐3′. Analysis of the qRT‐PCR data was performed by a ∆∆Ct method.^29^

### 2.5 Immunoblotting

Protein analysis by immunoblotting was performed as previously described.^24^ Antibody dilutions were as follows: 1:200 ASNS mouse monoclonal antibody,^30^ 1:1000 FLAG primary antibody (Cell Signaling no. 147935), 1:5000 antimouse IgG HRP‐conjugated secondary antibody (Bio‐Rad no. 170–6516), and 1:10 000 antirabbit IgG HRP‐conjugated secondary antibody (Bio‐Rad no. 170–6515).

### 2.6 Gas chromatography–mass spectrometry analysis of Asn

LCL cells were placed in T‐25 flasks upright at 1 × 10^6^ cells per ml in 10 ml RPMI ± Asn per replicate. After 24 h, the media were collected, cleared by centrifugation, and stored frozen at −80°C until processing. The cells were washed with cold 0.9% saline, then flash frozen in liquid nitrogen, and stored at −80°C as well. Cell pellets and media samples were processed and analyzed by gas chromatography‐mass spectrometry (GC‐MS), as reported previously.^24^, ^31^, ^32^ GC‐MS data acquisition was accomplished with a Thermo Scientific Single Quadrupole Mass Spectrometer (ISQ) and Gas Chromatograph (Trace 1310). Amino acid peak areas were processed with XCalibur Quan Browser software and normalized to the peak area of a DL‐norleucine internal standard. Asn concentrations were quantified with an external calibration curve.

### 2.7 Cell proliferation analysis

For cell growth analysis, a 2 ml aliquot of LCL wash plated at 5 × 10^4^ cells/ml per well in 12‐well plates and JRS cells were plated at 5 × 10^4^ cells per well in 6‐well plates. Following 0–96 h incubations in medium with or without Asn (RPMI for LCL, DMEM for JRS cells), cells were collected to determine viable cell numbers using the Applied Biosystems Countess II FL automated cell counter per the manufacturer's trypan blue staining protocol. All reported values are trypan‐excluding, viable cells only.

### 2.8 Purification of ASNS proteins from FLAG‐ASNS expressing HEK293T cell lines

The C‐terminal FLAG‐tagged WT ASNS expression construct was obtained from Sino Biological (no. HG16454‐CF) and then mutated by Genscript to obtain variant constructs for c.614A > C (p.H205P) and c.1192dupT (p.Y398Lfs*4), while retaining the FLAG‐tag. Variant and WT plasmids were transfected into HEK293T cells with X‐tremeGENE 9 transfection reagent (Roche no. 06365787001) and stable lines were selected in DMEM containing 100 μg/ml hygromycin B (EMD Millipore no. 400052‐5Ml) for at least 14 days, and then maintained in 50 μg/ml hygromycin B. ASNS‐FLAG expression was confirmed for each cell line by immunoblotting with anti‐FLAG antibody. FLAG‐tagged ASNS proteins were purified with EZview Red anti‐FLAG M2 Affinity Gel (Millipore Sigma no. F2426) as described.^24^ The purity of each protein preparation was confirmed by SDS‐PAGE and Coomassie Brilliant Blue R‐250 staining (Bio‐Rad 1 no. 61–0400) and aliquots were stored at −80°C until thawed only once for enzyme activity analysis.

### 2.9 AMP detection to monitor ASNS enzyme activity

As described previously,^24^ the AMP‐Glo assay kit (Promega no. V5011) was used to measure the AMP produced by purified WT or H205P ASNS protein per the manufacturer's protocol. Each assay was performed in triplicate to establish variability and each experiment was repeated at least once with a new protein preparation to establish reproducibility.

### 2.10 Differential scanning fluorimetry

Purified WT and H205P ASNS proteins were concentrated in Tris‐buffered saline (TBS, 30 mM Tris pH 7.6, 200 mM NaCl) using Amicon centrifugal concentrators (Sigma no. UFC201024). Samples for differential scanning fluorimetry (DSF) analysis were prepared with 2.5 μg of ASNS protein, 2.5 μl of 50 × SYPRO Orange (Invitrogen no. S6651 diluted in nuclease‐free water), and diluted to a 25 μl final volume with TBS. Following the Bio‐Rad Protein Thermal Shift protocol (Bulletin 7180) and using a Bio‐Rad CFX‐Connect Real‐Time System, the melting point of each protein was analyzed under cycling parameters of 10°C–95°C with increments of 0.5°C every 10 s and fluorescence detected after every increment.

### 2.11 Retroviral infection of ASNS‐null JRS cells

The ASNS‐FLAG open reading frame from the FLAG‐tagged ASNS expression construct (Sino Biological, no. HG16454‐CF) was subcloned into the MSCV‐PIG (Puro IRS green fluorescence protein [GFP]) expression plasmid (Addgene no. 18751) using Genscript. The resulting ASNS‐MSCV‐PIG retroviral expression plasmid was then mutated using Genscript to generate the c.614A > C and c.1192dupT mutant ASNS sequences within independent plasmids. Each construct retained the FLAG‐tag at the C‐terminal before the stop codon. The MSCV‐PIG plasmid encodes for puromycin selection and expresses IRES‐driven GFP as a method to measure transfection efficiency. Each construct was then packaged into retrovirus using HEK293T cells, and the resulting virus was used to infect JRS cells as previously reported.^24^ After infection, each WT or variant‐expressing cell line was selected in DMEM containing 1 μg/ml puromycin changed every 24 h for 72 h total. Expression was confirmed by GFP fluorescence and immunoblotting for ASNS.

### 2.12 Statistical analysis

All experimental data, except for immunoblots, represent assays in triplicate for a given experiment to determine variability. Every experiment was repeated at least once with an independent batch of cells to assess reproducibility. The data are shown as the averages ± SD and were analyzed by Student's two‐tailed *t*‐test. An asterisk represents a *p*‐value of at least ≤0.05 relative to the appropriate control for that experiment.

## 3 RESULTS

### 3.1 Clinical assessment

The male patient's maternal ancestry is of Cuban origin and paternal ancestry is of Italian origin. Family history was noncontributory. His perinatal history was notable for an increased nuchal thickness without polyhydramnios on prenatal ultrasound and follow‐up chorionic villus sampling ruled‐out aneuploidy. He was born at 39 and 4/7 weeks gestation via an uncomplicated spontaneous vaginal delivery and newborn hearing and metabolic screening were normal. At birth, his head circumference was 34 cm (z‐score = −0.89), and now, at age 4 years old, is 46.5 cm (z‐score = −3.31). Birth height and weight were unremarkable, 49.5 cm (z‐score = −0.17) and 3.74 kg (z‐score = 0.40), respectively. At 4 years of age, his height (101.7 cm, z‐score = −0.23) and weight (16.6 kg, z‐score = 0.03) continue to be appropriate for age. The patient presented for evaluation at the age of 9 months due to gross motor delays. His first seizure episode was at the age of 11 months in the context of a febrile illness due to adenovirus. This initial seizure was focal with unilateral weakness but was followed by subsequent generalized seizures in the setting of febrile illnesses. His seizures are now controlled by treatment with levetiracetam, at a dose of 1000 mg/d at age 4 years. No other seizure medications have been required. The initial routine electroencephalogram (EEG) was normal, but a subsequent 36‐h video EEG was abnormal with diffuse slowing, intermittent rhythmic slow waves and sharp waves. EEG patterns may vary in patients with ASNSD, but disorganization and intermittent sharp waves have been reported consistently. He is easily startled and develops jitteriness during intercurrent illnesses. Brain MRI at 11 months of age revealed parenchymal volume loss with otherwise normal anatomy.

The patient has global developmental delay. He rolled over at 6 months of age, sat at 11 months of age, and walked independently at 2.5 years of age. A developmental evaluation completed at 30 months of age revealed that his cognitive/play skills were at approximately a 13‐month‐old developmental level, while his gross and fine motor skills corresponded to a 14‐month and 11‐month‐old developmental level, respectively. Slow developmental progress in his motor skills was noted. He was able to walk without support and throw a ball, but unable to walk up or down stairs. His expressive and receptive language skills corresponded to an ~10 month and 8‐month‐old developmental level, respectively. The patient was able to produce some consonant‐vowel combinations but could not verbalize single words. He could respond to his name but was unable to follow basic instructions. Concerns were also raised regarding difficulties with attention and behavior regulation. Although delays were seen across all domains, there was no evidence at this time that these were the result of loss of skills or developmental regression. The patient also has mild bilateral myopic astigmatism and pseudo strabismus requiring glasses. Attempts at audiology evaluation have been unsuccessful. Cardiology (electrocardiogram and echocardiogram) evaluations were normal. The patient's weight gain and linear growth have been stable, but physical exam is notable for hypotonia, absence of spasticity, hyperreflexia, and microcephaly, which are consistent with ASNSD.

The diagnosis of ASNSD was made after NGS revealed that the patient has a paternally inherited (c.1192dupT) mutation and a maternally inherited (c.614A > C) mutation, that result in predicted pathogenic (p.Y398Lfs*4) and likely pathogenic (p.H205P) variants, respectively. The frameshift variant is predicted to result in nonsense‐mediated decay of the mRNA. The p.H205P variant is predicted to be deleterious by several in silico models and is present at a very low allele frequency in control populations (allele frequency of 0.000003778).^33^ Plasma amino acid levels, including Asn, were normal. Due to risks associated with sedation, the family declined further imaging or cerebrospinal fluid (CSF) evaluations.

### 3.2 Computational modeling of the reported variants

**FIGURE 1:**
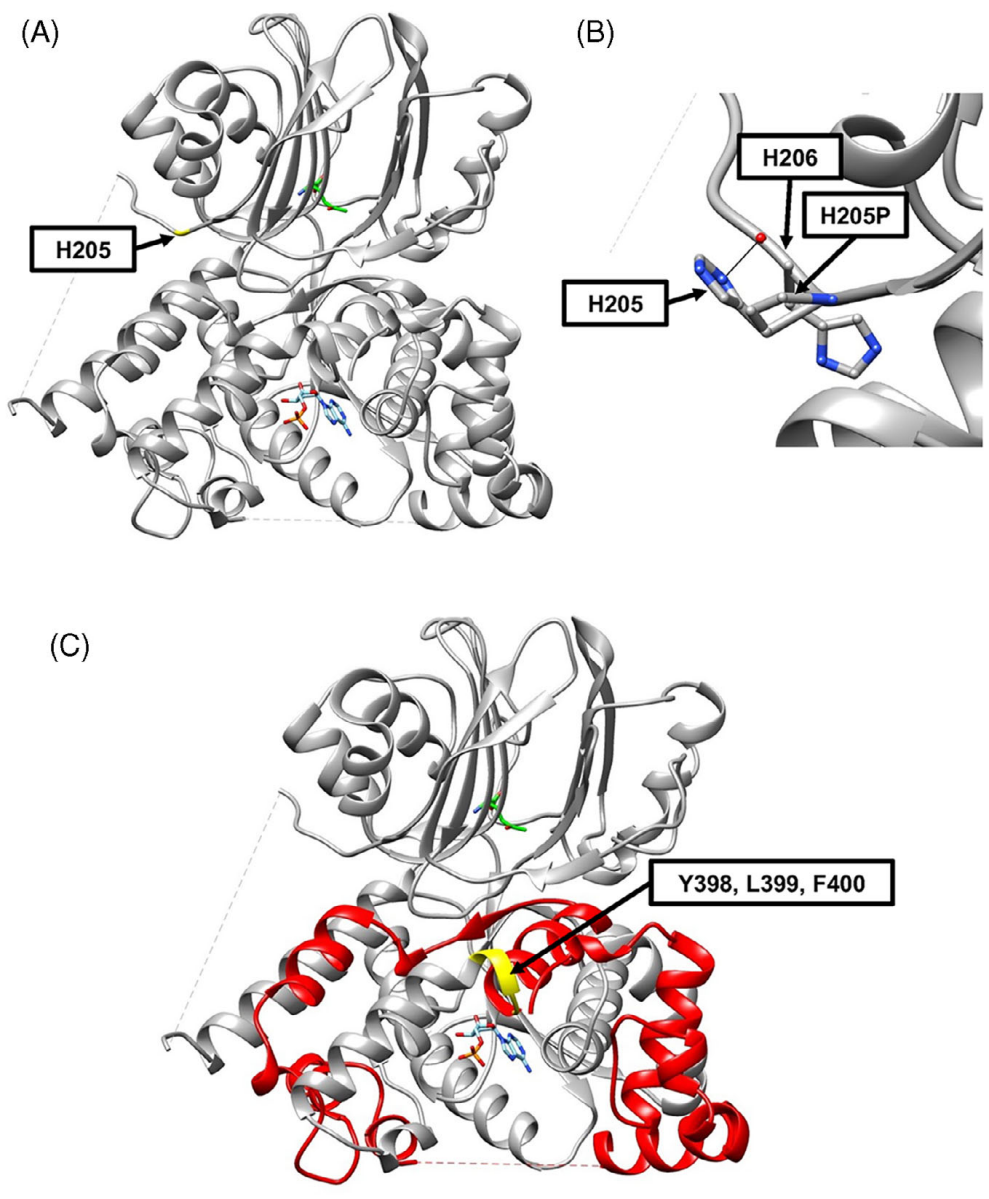
In silico modeling of variants within the asparagine synthetase (ASNS) protein structure. (A) The location of H205 in the context of the entire human ASNS crystal structure (PDB 6GQ3). H205 is shown in yellow, and the glutamine and AMP ligands are displayed in their binding pockets, with the AMP overlayed from the *Escherichia coli* AS‐B crystal structure (PDB 1CT9). (B) Hydrogen bonding is predicted between H205 and H206. No hydrogen bonding is found between the H205P most probable rotamer and nearby residues. (C) The Y398, L399, and F400 residues altered by the Y398Lfs*4 variant are highlighted in yellow while the subsequent residues shown in red are lost due to the premature stop codon introduced by the 1192dupT mutation. The AMP ligand is shown in its binding pocket as overlayed from the *E. coli* ASNS‐B crystal structure. For all ligands shown, nitrogen atoms are shown in blue, oxygen atoms in red, and phosphorous atoms in orange.

Computational modeling by introduction of ASNSD variants into the human ASNS crystal structure (PDB: 6GQ3)^6^ provides insight into the variant‐specific structural consequences in the context of the entire ASNS protein. Overlaying the *E. coli* AS‐B crystal structure (PDB: 1CT9), which identified the AMP binding site,^7^ shows that the H205 residue is localized within the N‐terminal domain with a minimum distance of 20 Å from the glutamine‐binding active site, as illustrated in Figure 1A. H205 is in a sequence that is believed to be an intrinsically disordered segment, that includes residues V210 to F223, linking the N‐terminal and C‐terminal domains (Figure 1A). Assessment of the hydrogen bonding of the H205 sidechain revealed only one predicted 2.9 Å hydrogen bond with the adjacent H206 residue (Figure 1B). Analyzing the most probable rotamer of H205P suggested loss of this H205 hydrogen bond because of an increase in the distance to 5.5 Å between H206 and H205P.^26^ The H205P rotamer does not appear to cause any steric clashing with nearby existing residues. Thus, the most likely explanation for any deleterious effect of H205P is the loss of hydrogen bonding leading to localized structural change, as well as the electrostatic and possible protonation change from histidine to proline.

The Y398Lfs*4 ASNS variant is more difficult to model because the premature stop codon causes truncation and loss of nearly half of the C‐terminal domain. The lack of computational tools to accurately predict protein folding limits the ability to model the impact on the remaining portion of the protein. To represent the Y398Lfs*4 variant, Figure 1C shows the residues in yellow that will be missense variants Y398L, L399F, and F400V, due to the shift in the codon reading frame caused by the 1192dupT nucleotide at the genomic level (Figure 1C). The resulting loss of residues D401‐A561 due to the early stop codon is illustrated in red. Clearly, the loss of these residues will alter the folding of the C‐terminal domain, binding of ligands in the C‐terminal domain, and, likely, the protein's overall stability.

### 3.3 Endogenous ASNS mRNA and protein levels in patient LCL

**FIGURE 2:**
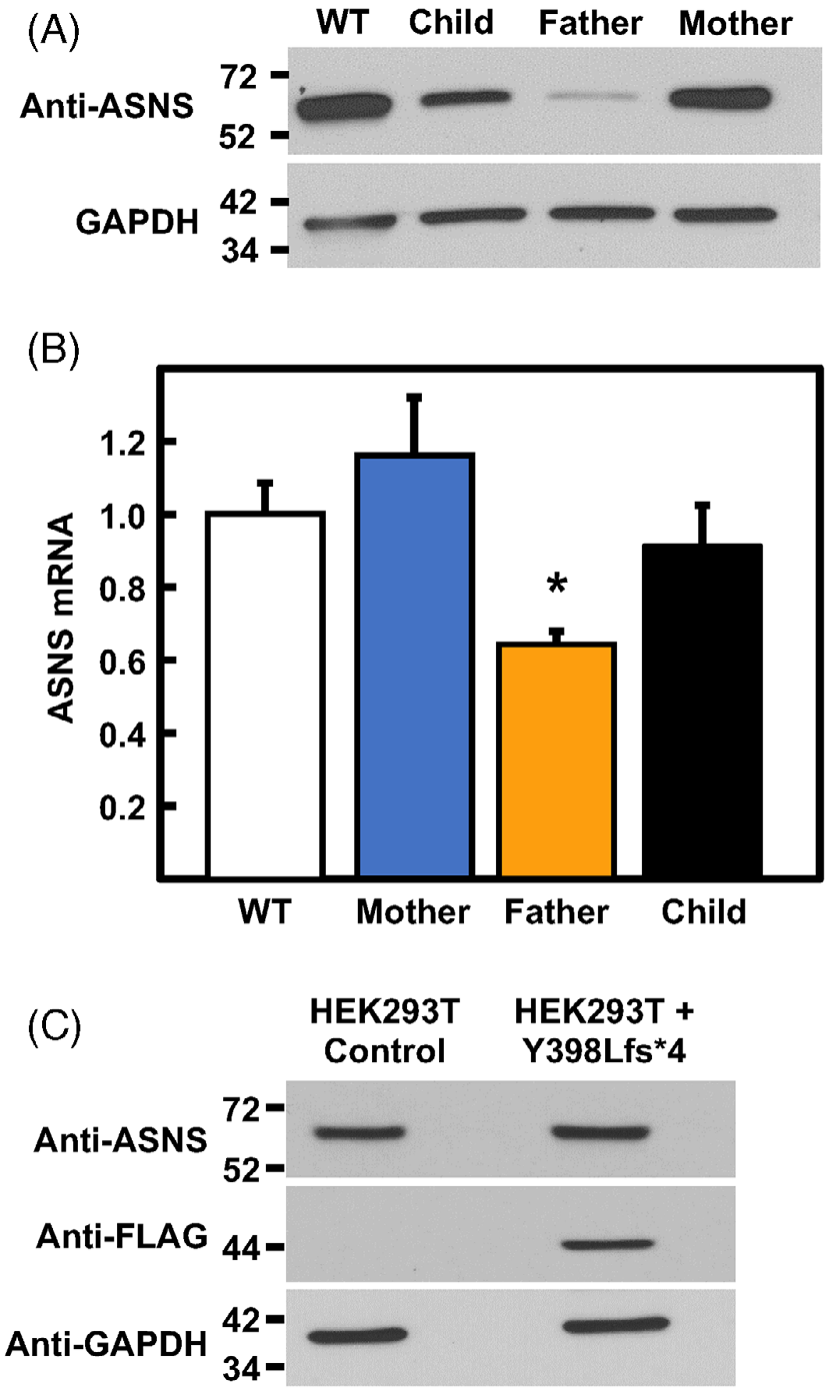
Asparagine synthetase (ASNS) expression in cultured lymphoblastoid cell lines (LCL) from family with asparagine synthetase deficiency (ASNSD). (A) ASNS protein expression measured by immunoblot of unrelated wild type (WT) and ASNS Deficiency (ASNSD) family‐derived LCL lysates. (B) ASNS mRNA measured by qRT‐PCR of total cellular RNA isolated from WT and family‐derived LCL. ASNS mRNA was normalized to GAPDH mRNA. The data are represented relative to the WT mRNA value set as 1 and are the averages ± SD in triplicate. An asterisk signifies a *p* ≤ 0.05 relative to WT. (C) The FLAG‐tagged Y398Lfs*4‐encoding construct was transfected in to HEK293T cells and stably expressing cell lines were selected. After selection, cell extracts were subjected to immunoblotting for endogenous ASNS, FLAG‐tagged Y398Lfs*4, and GAPDH.

The computational modeling of both ASNS variants suggested possible protein instability. ASNS immunoblot analysis of LCL protein extracts from an unrelated WT and the family showed relatively similar expression in the WT and maternal cell lines, a detectably lower level in the child, and a substantially reduced ASNS abundance in the paternally derived cells (Figure 2A). To investigate whether the reduced ASNS expression in the paternal LCL was reflected by mRNA abundance, the ASNS steady‐state mRNA levels were determined by qRT‐PCR. Among the family, only the paternal LCL showed a significant reduction in ASNS mRNA levels (Figure 2B), consistent with a prediction of nonsense‐mediated decay for the mRNA encoding this mutation. These observations were reproducible across RNA isolations from different batches of cells. Interestingly, neither the child nor the paternal cell line showed any ASNS expression at a lower molecular mass than WT ASNS that might be indicative of stable expression of the truncated Y398Lfs*4 variant (data not shown). Although the epitope specificity of the anti‐ASNS monoclonal antibody used to obtain the data presented in Figure 2A is unknown, an N‐terminal‐specific anti‐ASNS antibody was also tested and failed to detect a truncated protein (data not shown). To investigate the possibility that the truncated ASNS variant may lead to degradation of the WT protein the father produces from his other allele, the FLAG‐tagged Y398Lfs*4 variant was ectopically expressed in HEK293T cells and the level of ASNS measured by immunoblotting (Figure 2C). In two independent experiments, it was observed that, using the FLAG antibody, a truncated ASNS protein of about 44 kDa could be readily detected and that expression of the truncated protein had no deleterious effect on the amount of endogenous full‐length HEK293T ASNS, as detected with anti‐ASNS antibody.

### 3.4 Enzyme activity and thermal stability of purified ASNS proteins

**FIGURE 3:**
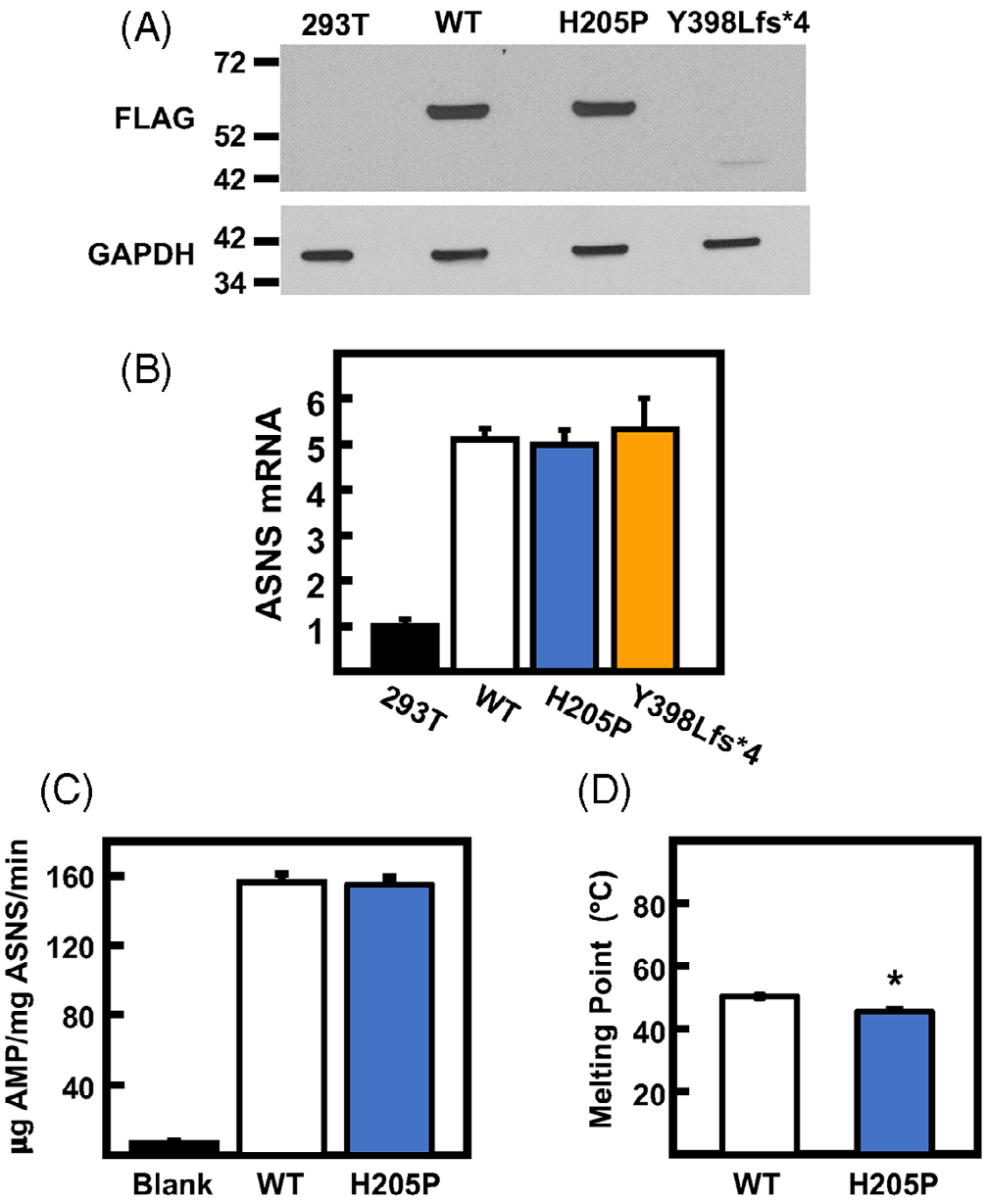
Enzyme activity and stability of purified asparagine synthetase (ASNS) proteins. (A) Immunoblot of FLAG‐tagged ASNS protein in HEK293T cells stably expressing wild type (WT), H205P, or Y398Lfs*4 proteins. Protein extracts, including that from a nontransfected HEK293T cell line, were probed with anti‐FLAG antibody. (B) ASNS mRNA content was established for each of the cell lines and normalized to GAPDH mRNA. The data are represented relative to the control nontransfected HEK293T value set as one. (C) Extracts from the WT and H205P variant‐expressing HEK293T cell lines were used to measure ASNS enzymatic activity. A negative control reaction with no protein added was used as a reference of the assay background value (Blank). (D) Thermal stability was evaluated through melting point detection of purified WT and H205P ASNS protein. For Panels B–D, the data represent averages ± SD in triplicate with *p ≤ 0.05 relative to WT.

An approach to independently express and evaluate the enzymatic activity of the H205P and Y398Lfs*4 variants compared with WT was initiated by stable expression of each FLAG‐tagged protein in HEK293T cells. After stable cell lines were established, the level of FLAG‐ASNS expression was determined by immunoblot using FLAG antibody (Figure 3A). The un‐transfected HEK293T cells showed no FLAG signal as expected, whereas the FLAG‐WT and FLAG‐H205P expressing cells showed an abundance of ASNS protein at the expected 64 kDa molecular mass. For the HEK293T cells stably expressing the FLAG‐Y398Lfs*4 protein, a band at ~45 kDa was faintly visible on long exposure of the film, but the amount was near the limit of detection (Figure 3A). To ensure that the FLAG‐Y398Lfs*4 cell line stably expressed the cDNA, the mRNA levels were determined by qRT‐PCR. The results showed elevated expression for all stable cell lines above the baseline ASNS mRNA levels in untransfected HEK293T cells (Figure 3B). Cell extracts were subjected to FLAG affinity‐based purification to obtain pure ASNS proteins. For the WT and H205P variant abundant protein was purified, but purification of detectable amounts of the Y398Lfs*4 protein could not be achieved. The enzymatic activity of WT and H205P ASNS proteins showed no significant difference (Figure 3C). The melting temperature of both proteins was determined by a DSF. The H205P variant showed a small, but statistically significant 4.8°C decrease in melting temperature compared with the WT ASNS protein (Figure 3D).

### 3.5 GC‐MS analysis of Asn content

**FIGURE 4:**
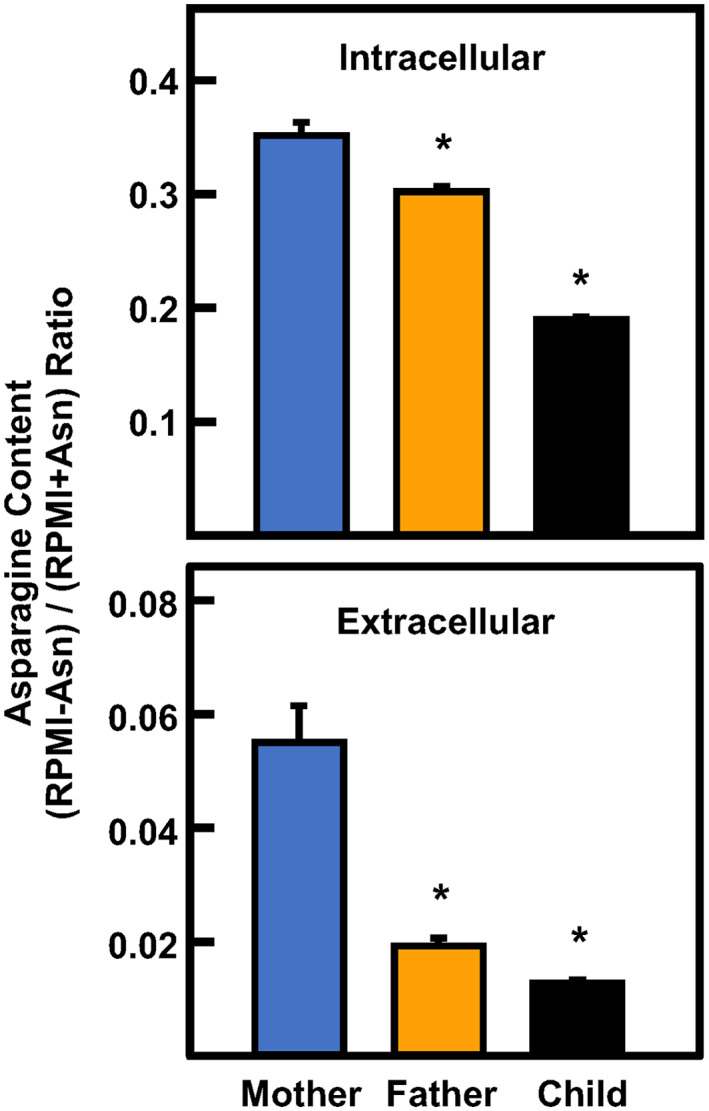
Intracellular and extracellular asparagine content in asparagine synthetase deficiency (ASNSD) lymphoblastoid cell lines (LCL). Gas chromatography–mass spectrometry analysis for intracellular and extracellular asparagine (Asn) was performed after incubation of LCL for 24 h in RPMI medium with or without Asn. The Asn concentration was calculated as ng/μl/10^6^ cells. To best illustrate the effect of Asn deprivation, the results are presented as the ratio of the values obtained in the two conditions, (RPMI − Asn)/(RPMI + Asn). The data represent averages ± SD in triplicate and an asterisk indicates a *p* ≤ 0.05 relative to the values obtained for the mother's cells.

To test the impact of ASNS variant expression on Asn content in the family's LCL, the cells were incubated in RPMI ± Asn for 24 h and then subjected to GC‐MS analysis (Figure 4).

The Asn concentrations were calculated for each family member and are presented as the ratio from the two media (RPMI − Asn)/(RPMI + Asn). The values for both intracellular and extracellular Asn were the highest for the mother (Figure 4), perhaps a reflection that the H205P variant has enzymatic activity nearly identical to WT enzyme (Figure 3C). The results show that the intracellular Asn content of the father's cells was modestly, but significantly reduced relative to the mother's value and this decrease was even greater for the child (Figure 4). The 24 h incubation medium was also analyzed for Asn and revealed an even more striking pattern. Relative to Asn‐replete medium, after incubation in the Asn‐free medium the extracellular Asn content of the father's cells was reduced by 65% of the value for the mother. For the child, the extracellular Asn content ratio was reduced by about 75% relative to the mother's value. This observation that the decline in extracellular Asn appears to better reflect the degree of ASNS activity loss than does the intracellular Asn content is consistent with our previous observations for fibroblast studies from two independent ASNSD families.^24^

### 3.6 Impact of Asn availability on proliferation of ASNSD LCL

**FIGURE 5:**
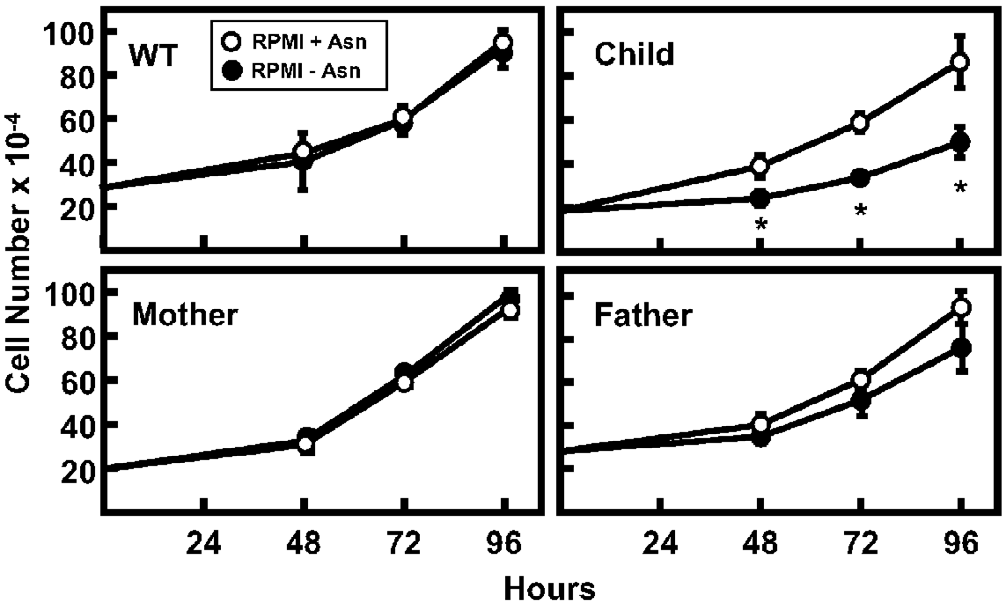
Impact of asparagine synthetase (ASNS) variant expression on ASNS deficiency (ASNSD) lymphoblastoid cell lines (LCL) proliferation. Proliferation of unrelated WT and family‐derived LCL was analyzed during culture in RPMI medium with or without asparagine (Asn) for 0, 48, 72, or 96 h. The data represent averages ± SD in triplicate and an asterisk indicates a *p* ≤ 0.05 relative to the value obtained in the presence of Asn.

Evaluation of LCL proliferation in the presence or absence of extracellular Asn revealed that the WT LCL showed no difference, as expected for a cell with endogenous ASNS activity (Figure 5). Under the same conditions, the maternal‐derived cell line showed similar proliferation at all timepoints, whereas the paternal LCL showed a reproducible trend of decreased proliferation at the 72 and 96 h time points when maintained in medium lacking Asn. LCL from the ASNSD child showed a significant reduction in proliferation when cultured in Asn‐free medium for 48 h or more (Figure 5). Given that this is the first report of ASNSD analysis using LCL cells, it is reassuring to observe results consistent with our previously reported analyses of primary skin fibroblasts from multiple ASNSD families.^22^, ^23^, ^24^ In this case, the child's cells do show some proliferation in the absence of Asn, suggesting that at least one of the variants has sufficient ASNS activity to support partial growth. This interpretation at the cellular level is consistent with the enzymatic assay of purified protein showing that the H205P variant exhibits little or no decline in activity (Figure 3C).

### 3.7 Evaluation of individual variants by expression in ASNS‐null cells

**FIGURE 6:**
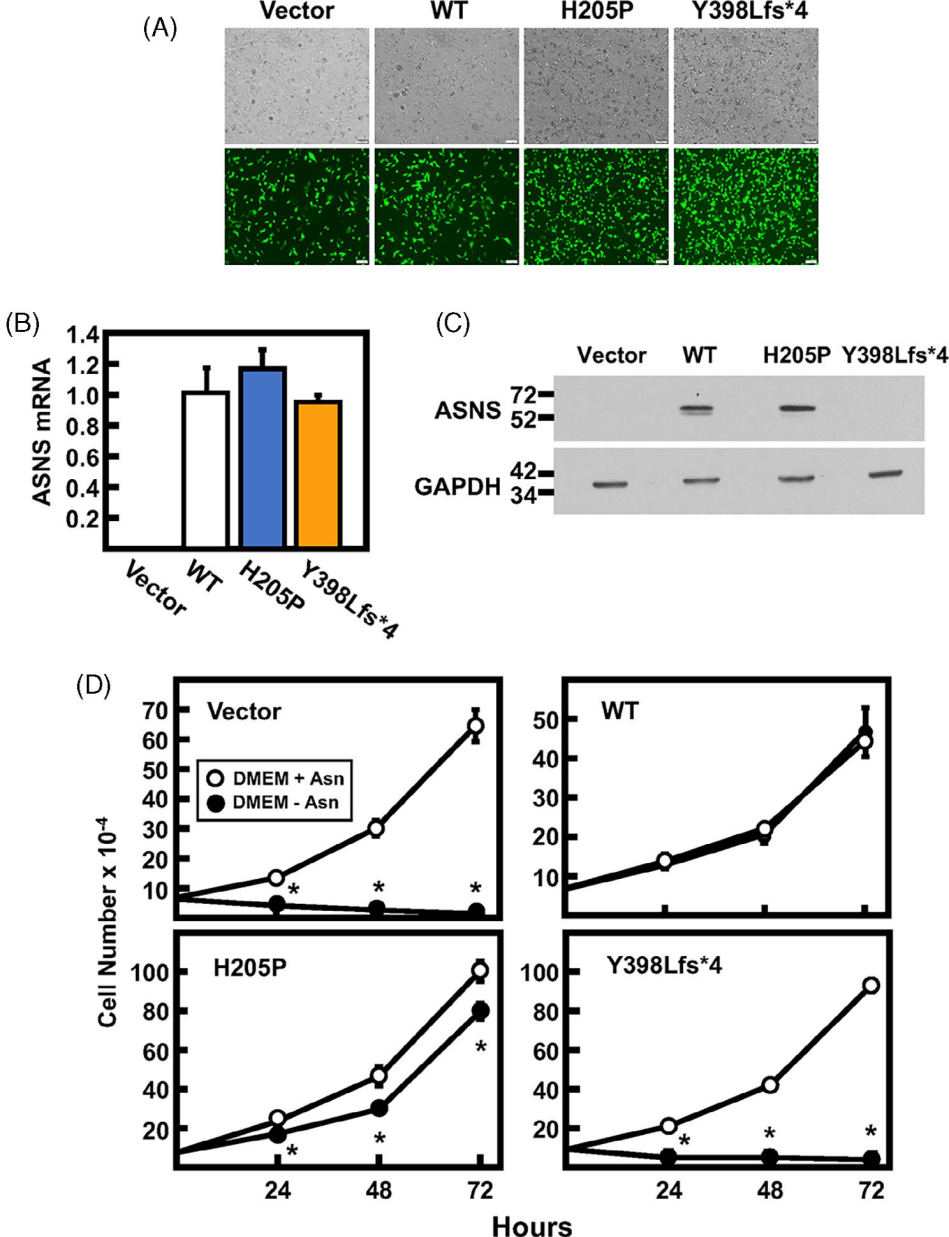
Impact of asparagine synthetase (ASNS) variant expression in ASNS‐null cells. (A) An ASNS‐null cell line (Jensen Rat Sarcoma, JRS) was transduced with either an empty “vector”, wild type (WT), H205P, or Y398Lfs*4 ASNS‐expressing retrovirus that also co‐expressed green fluorescent protein (GFP). Brightfield (BF) images (top panels) were taken and GFP was detected (bottom panels) for each cell line to evaluate the relative transduction efficiency. (B) ASNS mRNA was detected by quantitative real‐time PCR for each JRS cell line, the data were normalized to GAPDH and are presented relative to the WT level set to one. (C) Immunoblot detection of ASNS in stably expressing JRS cells. (D) Cell proliferation was measured in JRS cells stably expressing the indicated ASNS variant after incubation for 0, 24, 48, or 72 h in DMEM with or without Asn. For Panels B and D, the data represent averages ± SD in triplicate and an asterisk indicates a *p* ≤ 0.05 relative to the control.

To investigate the impact of each mutation in the absence of a second allele, each variant was ectopically expressed in JRS cells, an ASNS‐null cell line.^34^ A second advantage of this approach is that JRS cells provide a uniform genetic background, in contrast to independently prepared and selected LCL from each family member. In JRS cells, hypermethylation of the endogenous ASNS gene results in no detectable ASNS expression. JRS cells transduced with a retrovirus lacking an ASNS insert (“Vector,” GFP expression only) or retrovirus vector with WT, H205P, or Y398Lfs*4 expression constructs were used to generate stably expressing cell lines. The transduction efficiency and uniformity were evaluated by fluorescence microscopy for the co‐expressed GFP (Figure 6A) and confirmed by qRT‐PCR for ASNS mRNA content (Figure 6B). There were no significant differences in ASNS mRNA levels among the three mutants expressed in the JRS cells, whereas the “vector only” JRS cells showed no detectable ASNS mRNA, as expected. Assessment of variant protein expression by immunoblotting with anti‐ASNS antibody showed a similar abundance for the WT and H205P cell lines, but no expression of ASNS in the stable Y398Lfs*4 cell line (Figure 6C). The paternal LCL (Figure 2C) and the Y398Lfs*4‐expressing JRS (Figure 6C) cell lines do not exhibit a detectable truncated ASNS band, whereas expression of the Y398Lfs*4 construct in HEK293T cells yielded a visible truncated protein (Figure 3A). The simplest explanation for this observation is a cell‐specific difference in protein turnover, which allows the Y398Lfs*4 truncated protein to accumulate in HEK293T cells. Using the stably expressing JRS cells, tests for proliferation in the absence of medium‐supplied Asn revealed that WT ASNS cells showed no significant difference between growth in the presence or absence of extracellular Asn (Figure 6D). In contrast, when cultured in the absence of Asn, both the vector only JRS cells and the Y398Lfs*4 cells exhibited considerable cell death over the 72‐h monitored. The H205P cells showed about a 20%–30% reduction in proliferation in the absence of Asn (Figure 6D).

## 4 DISCUSSION

Clinically, the male child characterized here presented with seizures, postnatal microcephaly, and global developmental delays. While he is severely affected, his motor skills are more advanced than most previously reported ASNSD cases, indicating that ASNSD has a wide clinical spectrum and should be considered in the differential diagnosis of seizures and developmental delay. The majority of patients with ASNSD reported to date have suffered from severe epilepsy and died in infancy or early childhood. However, recent cases highlight that some individuals with milder missense variants may have more attenuated developmental delay and longer survival.^21^ This case further expands the phenotypic spectrum of ASNSD, as the patient continues to make developmental progress at 4 years of age and his seizures are well‐controlled on levetiracetam monotherapy. A full understanding of genotype–phenotype correlations in ASNSD is lacking. Functional analysis of these mild cases may help elucidate these relationships.

To the best of our knowledge, the present report includes the novel use of immortalized LCL prepared from a child and parents having ASNSD. We have used primary fibroblasts in previous descriptions of three families with ASNSD,^22^, ^23^, ^24^ but we view the use of LCL as an important technical development that will promote additional ASNSD research. Obtaining lymphocytes from a blood draw is likely to be acceptable to more parents than skin biopsies. Also, LCL is amenable to a wider range of research protocols than human fibroblasts and, because they are grown in suspension, they can be more readily obtained in much larger quantities. Beyond the first use of LCL, this report expands ASNSD knowledge by documenting a child who has the biallelic mutations c.614A > C (maternal) and c.1192dupT (paternal), which lead to the ASNS variants p.H205P and p.Y398Lfs*4, respectively. The current report intertwines the clinical description of the patient with biochemical and cellular data encompassing characterization of Asn metabolism in patient‐derived cells in culture, enzyme activity analysis, and a functional evaluation of each variant after stable expression in ASNS‐null cells.

The present mutations and resulting variants expand the landscape of ASNSD and highlight the distribution across the entire protein structure.^21^ Computational modeling of the H205P variant for the present family revealed its location away from the active site, within a linker region, and near a disordered segment on the exterior of the protein. There was no obvious structural impact of introduction of the most probable H205P rotamer besides the loss of a single hydrogen bond with the adjacent residue and a change in electrostatic potential. However, the in vitro data does not indicate a high degree of instability, because when expressed in the HEK293T cells the H205P protein's abundance and purification were similar to the WT ASNS protein. In contrast, the Y398Lfs*4 variant has an obvious structural change given the large truncation induced by the newly encoded stop codon that results in the loss of a substantial portion of the C‐terminal domain. It is likely that this variant is unstable, an interpretation that is supported by the observations that ectopic expression in both HEK293T and JRS cell types yielded little to no detectable protein and purification was not possible.

ASNSD diagnosis involves panel‐based or exome sequencing, which can identify possible pathogenic mutations within the coding region of the *ASNS* gene. What is lacking in these analyses are possible mutations in transcriptional control sequences of this highly regulated gene that is responsive to nutrient availability and many other cellular stress signals.^9^, ^10^, ^11^, ^35^ For example, ASNS is transcriptionally regulated by ER stress^36^, ^37^ or by cellular deprivation for any single limiting amino acid.^38^, ^39^, ^40^ To indirectly test for possible noncoding mutations that would alter basal ASNS expression, we have tested for mRNA and protein abundance in the LCL of each family member. The ASNS mRNA expression in the mother's cells was similar to an unrelated WT cell line, as was the child's, although the child's total protein level was reduced. In contrast, despite the fact that he has a WT allele, the father's ASNS mRNA and protein levels were clearly decreased. Computer analysis of the Y398Lfs*4 mutation suggests that the corresponding mRNA may be subject to nonsense‐mediated decay. Another possibility, not mutually exclusive, is that the father's genome has mutations that affect transcription from the *ASNS* gene. We questioned whether or not the sharply reduced protein abundance in the father resulted from the truncated protein having a negative effect on the abundance of the ASNS protein produced from the WT allele. However, when the truncated protein was over‐expressed in HEK293T cells, there was no decrease in the full‐length ASNS protein abundance. Consequently, the molecular basis for the reduction in father's ASNS mRNA and protein levels must await further mechanistic investigation.

To understand the metabolic impact of mutations in the *ASNS* gene, it is essential to determine the enzymatic activity and stability of each ASNS variant. To evaluate the present family, each variant was expressed in HEK293T cells and purified. To exclusively exhibit glutamine‐dependent activity, mammalian ASNS is posttranslationally processed to remove the N‐terminal methionine which generates a mature protein with an N‐terminal cysteine, initially present at the second position. This processing event does not occur after expression of the mammalian protein in bacterial cells, thus there is a need for ectopic expression in mammalian cells prior to purification for enzymatic assay.^2^, ^6^ Despite abundant mRNA after transfection and selection of HEK293T cells, the truncated FLAG‐Y398Lfs*4 protein was at or below the detection limit by anti‐FLAG immunoblotting and no protein could be purified for assay. These results are consistent with the hypothesis that the protein may not be stable. Conversely, the WT and H205P proteins could be readily purified and the H205P variant showed no decrease in activity, consistent with the structural modeling that H205 is within an unstructured region of the protein and there is little effect of the H205P substitution on the overall ASNS structure. This interpretation agrees with the observation that the LCL derived from the mother exhibited little or no reduction in proliferation in the absence of Asn. However, this result contrasts with many other ASNS variants assayed by our laboratory (unpublished results) and by others^6^, ^20^ that have shown reduction in enzyme activity. Analysis of intracellular and extracellular Asn concentrations, after incubation of the LCL in RPMI medium ± Asn, revealed that the cells from the father and the child were not able to maintain Asn levels nearly as well as the mother's cells.

In the study of inborn errors of metabolism, it is foundational to assess disease‐associated mutations independent of the patient's genetic background, as recently reported by us for two variants^24^ and by others for four independent ASNS variants.^20^ We tested the impact of the H205P and Y398Lfs*4 proteins in ASNS‐null JRS cells, which provide a common genetic background and assessment independent of a second WT or mutated allele. The Y398Lfs*4 transfected cells showed a complete lack of proliferation, similar to the JRS cells transfected with an empty vector, whereas the H205P‐expressing cells showed a 20% reduction in proliferation. Thus, although the Asn‐independent growth of the maternal LCL cells and the enzymatic activity of the H205P protein were not detectably decreased, there was a reduction in proliferation when the H205P variant is solely responsible for cellular ASNS function.

To prevent excess ammonia accumulation in the brain, it is documented that transporters at the two surfaces of the endothelial cells that make up the blood–brain barrier coordinately function such that there is net flux of Asn, glutamine, and histidine from the CSF to the plasma.^41^ Thus, the brain relies heavily on intracellular Asn synthesis rather than dietary Asn from the circulation. It follows then that during development, when the blood‐brain barrier becomes functional and operates to remove Asn from the CSF, expression of ASNS variants with deficient activity or stability hamper the cellular proliferation within the developing brain and lead to the observed microcephaly and brain atrophy of these children. The present report describes two novel ASNS variants that expand our observations of impaired Asn metabolism due to reduced enzymatic activity or protein stability. The results provide additional support to the ever‐increasing knowledge base concluding that loss of ASNS activity contributes to the ASNSD phenotype of impaired development and function of both central and peripheral neurological systems. Future studies in animal models of ASNSD represent an excellent opportunity to identify the causal links between decreased ASNS activity and the anatomic, physiologic, and biochemical observations, both in the brain as well as in peripheral tissues.

## AUTHOR CONTRIBUTIONS

Stephen J. Staklinski, Mario C. Chang, Matthew E. Merritt, and Michael S. Kilberg designed and conceptualized the study. Rebecca C. Ahrens‐Nicklas, Shagun Kaur, and Arianna K. Stefanatos performed the clinical analysis and generated the LCL. Stephen J. Staklinski, Mario C. Chang, and Elizabeth E. Dudenhausen performed the biochemical and cell biological studies. Stephen J. Staklinski, Mario C. Chang, and Michael S. Kilberg analyzed the data and prepared the figures. Stephen J. Staklinski, Mario C. Chang, Rebecca C. Ahrens‐Nicklas, and Michael S. Kilberg wrote the initial article draft and all authors participated in revising the article and figures.

## FUNDING INFORMATION

Funding for Michael S. Kilberg was from the National Institutes of Health and Institute of Child Health and Human Development (HD100576).

## CONFLICT OF INTEREST

The authors have no known competing financial interests or personal relationships that influenced the work presented.

## Data Availability

All original data will be supplied upon request.
